# Unique Ca^2+^-Cycling Protein Abundance and Regulation Sustains Local Ca^2+^ Releases and Spontaneous Firing of Rabbit Sinoatrial Node Cells

**DOI:** 10.3390/ijms19082173

**Published:** 2018-07-25

**Authors:** Tatiana M. Vinogradova, Syevda Tagirova (Sirenko), Edward G. Lakatta

**Affiliations:** Laboratory of Cardiovascular Science, Intramural Research Program, National Institute on Aging, NIH, 251 Bayview Blvd, Room 8B-123, Baltimore, MD 21224, USA; syevda.tagirova@nih.gov (S.T.); lakattae@grc.nia.nih.gov (E.G.L.)

**Keywords:** cardiac pacemaker, sinoatrial node cells, SR Ca^2+^ ATP-ase (SERCA), phospholamban, ryanodine receptors

## Abstract

Spontaneous beating of the heart pacemaker, the sinoatrial node, is generated by sinoatrial node cells (SANC) and caused by gradual change of the membrane potential called diastolic depolarization (DD). Submembrane local Ca^2+^ releases (LCR) from sarcoplasmic reticulum (SR) occur during late DD and activate an inward Na^+^/Ca^2+^ exchange current, which accelerates the DD rate leading to earlier occurrence of an action potential. A comparison of intrinsic SR Ca^2+^ cycling revealed that, at similar physiological Ca^2+^ concentrations, LCRs are large and rhythmic in permeabilized SANC, but small and random in permeabilized ventricular myocytes (VM). Permeabilized SANC spontaneously released more Ca^2+^ from SR than VM, despite comparable SR Ca^2+^ content in both cell types. In this review we discuss specific patterns of expression and distribution of SR Ca^2+^ cycling proteins (SR Ca^2+^ ATPase (SERCA2), phospholamban (PLB) and ryanodine receptors (RyR)) in SANC and ventricular myocytes. We link ability of SANC to generate larger and rhythmic LCRs with increased abundance of SERCA2, reduced abundance of the SERCA inhibitor PLB. In addition, an increase in intracellular [Ca^2+^] increases phosphorylation of both PLB and RyR exclusively in SANC. The differences in SR Ca^2+^ cycling protein expression between SANC and VM provide insights into diverse regulation of intrinsic SR Ca^2+^ cycling that drives automaticity of SANC.

## 1. Introduction

**Figure 1:**
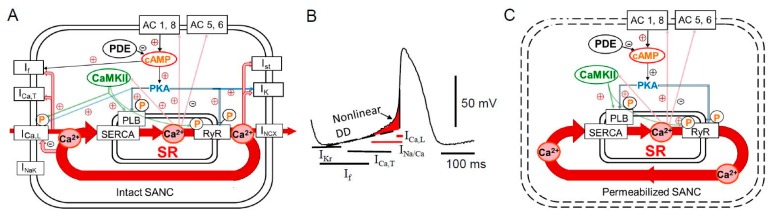
A simplified schematic illustration of regulation of the basal cardiac pacemaker function by Ca^2+^, and PKA- and CaMKII-dependent phosphorylation in intact and permeabilized rabbit SANC. (**A**) The coupled-clock pacemaker system. Intracellular Ca^2+^ cycling (in red) operates in tight cooperation with the ensemble of membrane ion channels. Note that L-type Ca^2+^ channel and I_NCX_ current are both Ca^2+^ cycling proteins and surface membrane currents. Constitutive activation of the basal AC activity results in an elevated level of cAMP and cAMP/PKA-dependent phosphorylation. The basal Ca^2+^/cAMP-PKA “feed-forward” regulation is kept in check by a high basal PDE activity. Many ion currents including (I_f_, I_st_, I_Ca,L_, I_Ca,T_, I_K_, etc.) are regulated by Ca^2+^. Both PKA- and CaMKII-dependent phosphorylation modulates the function of Ca^2+^ cycling proteins (PLB, RyR, L-type Ca^2+^ channel, and I_NCX_ current). (**B**) Schematic illustration of spontaneous SANC action potential and major currents involved in generation of the diastolic depolarization. See text for additional details. LCR-induced increase in [Ca^2+^] beneath sarcolemma activates an inward I_NCX_ current creating exponential increase in the diastolic depolarization rate. (**C**) Schematic illustration of permeabilized SANC in the absence of functional ionic currents. Ca^2+^ cycling by the SR in permeabilized SANC becomes “free running” and is controlled mostly by the concentration of free cytosolic [Ca^2+^]_f_ and the kinetics of Ca^2+^ pumping into SR and release from RyR. See text for additional details.

The heart beats over 3 billion times during a normal life-span. The sinoatrial (SA) node, the primary physiological pacemaker of the heart, is a specialized and relatively small area in the right atrium which spontaneously generates action potentials. Sinoatrial node cells (SANC) are able to generate spontaneous action potentials because of the gradual depolarization of the membrane potential during diastole, called diastolic depolarization. The ensemble of sarcolemmal ion channels and electrogenic ion transporters (including L- and T-type Ca^2+^ channels (I_CaL_ and I_CaT_, respectively), delayed rectifier K^+^ channels (I_K_), hyperpolarization-activated funny channels (I_f_), Na^+^/Ca^2+^ exchanger (NCX) current (I_NCX_), Na^+^/K^+^ ATPase current (I_NaK_), etc.) are involved in the generation of the diastolic depolarization (Figure 1A,B). Numerous reviews have described the contribution of ionic currents in the generation of the diastolic depolarization and cardiac pacemaker function [1,2,3,4].

Effects of changes in extracellular [Ca^2+^] on spontaneous beating rate of the intact SA node have also been well established in early studies [5,6], but its effect on cardiac pacemaking was originally explained by Ca^2+^-induced changes in several ionic currents including I_f_ [7], I_Ca,T_ and I_Ca,L_ [8], I_K_ [9,10], I_st_ [11], etc. Although the involvement of intracellular Ca^2+^ cycling in cardiac pacemaker function was studied over the next decade [12,13,14,15,16,17,18], specific mechanisms of intracellular sarcoplasmic reticulum (SR) Ca^2+^ cycling and their importance for spontaneous SANC firing were discovered in relatively recent studies and described in numerous reviews [19,20,21,22,23,24,25,26]. 

Similar to other excitable cardiac cells, SANC cycle Ca^2+^ into and out of their SR. Ca^2+^ influx via L-type channels during each spontaneous cycle induces global Ca^2+^ release from ryanodine receptors (RyRs), SR Ca^2+^ release channels, creating Ca^2+^-induced Ca^2+^ release (CICR) [27]. Furthermore, SANCs can generate spontaneous local Ca^2+^ releases (LCRs) that appear during late diastolic depolarization before the next action potential upstroke [19]. Since SANCs do not have T-tubules, LCRs occur beneath sarcolemma of SANC and activate a forward mode of the Na^+^/Ca^2+^ exchanger, generating an inward Na^+^/Ca^2+^ exchange current (I_NCX_), which accelerates the diastolic depolarization rate leading to earlier occurrence of the next action potential [19,28]. Numerous studies have confirmed presence of spontaneous rhythmic LCRs under normal physiological conditions in SANC of multiple species including humans [13,19,20,23,29,30,31,32]. LCRs are roughly periodic and could be suppressed by a rhizome alkaloid ryanodine [12,17,19,33], which locks RyR in subconductance open state, depleting the SR Ca^2+^ content [34]. 

Since SR-generated LCRs persist in the absence of sarcolemmal ionic function, i.e., in saponin-permeabilized rabbit SANC [23,35] or during voltage clamp [23], they occur spontaneously and were called intracellular “Ca^2+^ clock” [28] (Figure 1C). Ca^2+^ influx via L-type Ca^2+^ channels trigger a global SR Ca^2+^ transient (via CICR) that partially depletes SR, resets the “Ca^2+^ clock” and temporarily suspends generation of spontaneous LCRs [23,28]. The local Ca^2+^ release period is a time-interval from the action potential-triggered global Ca^2+^ transient to the occurrence of LCR. The LCR period defines the time of Ca^2+^ release beneath sarcolemma and activation of inward I_NCX_ [19]. The inward I_NCX_ produces an exponential increase in the diastolic depolarization rate leading to earlier occurrence of the action potential upstroke and thus regulates the spontaneous SANC beating rate [23,28,36]. The local Ca^2+^ release period is critically dependent on the rate of SR Ca^2+^ replenishment, controlled by SR Ca^2+^ ATP-ase (SERCA) [36]. 

During each spontaneous cycle, the intracellular “Ca^2+^ clock” in SANC dynamically interacts with the ensemble of surface membrane ion channels and other electrogenic carriers and pumps, the “Membrane clock”. Specifically, L-type Ca^2+^ channels and I_NCX_ control Ca^2+^ influx and efflux from the cell and define the amount of Ca^2+^ available for pumping into SR and, as a result, govern the SR Ca^2+^ load and timing of LCR generation (Figure 1A,B). The most recent simulations explored a possible positive feedback mechanism, called action potential ignition, which accelerates the diastolic depolarization to reach the action potential threshold [37]. Therefore, in intact SANC, the “Ca^2+^ clock” and “Membrane clock” function together and mutually entrain each other since changes in membrane currents directly or indirectly regulate intracellular Ca^2+^ cycling, while changes in SR Ca^2+^ cycling, in its turn, modulate parameters of ionic channels [24,25,26,28,38].

Subsequent research revealed the identity of additional players that are involved in the regulation of basal cardiac pacemaker function which added additional complexity to intrinsic signaling mechanisms in SANC, including phosphorylation cascades [39,40], calcium signaling through TRP [41,42], IP_3_ channels [43], Ca^2+^-dependent small conductance K^+^ channels [44] as well as mitochondria [45,46]. 

Other studies began to compare expression of ionic channels and Ca^2+^ handling proteins at the messenger RNA level between SA node and working myocardium of varied species including humans, rabbits and mice [47,48,49,50,51,52]. mRNA expression, however, could be inconsistent with the protein expression or functional data due to messenger instability, ratio of protein synthesis and degradation or post translational modification. For example, Kir2 channels are responsible for the inward rectifier K^+^ current (I_K1_), a key player in generating the resting membrane potential in working myocardium (atrial and ventricular myocytes). Although a similar mRNA expression pattern of Kir2.1 and Kir2.2 was observed in the SA node and atrium of adult rabbits [47], I_K1_ was present in rabbit atrial myocytes [53], but entirely absent in rabbit SANC [2]. 

The proteomics approach showed substantial differences in the expression of multiple proteins between human SA node and working myocardium [54]. These varieties might be based on different formation of the SA node and working myocardium, i.e., in the developing heart, the venosus pole consists of the sinus venous and the primitive atrium, which are separated by a sinoatrial fold [55,56]. The SA node expresses Tbx3 and Hcn4, whereas the atrium expresses Cx40 and Nppa in a complementary manner [57,58], suggesting that atrial and SA node lineages separate as soon as they start to express these markers [3,55]. However, despite the importance of Ca^2+^ handling proteins for cardiac pacemaker function, very little is known about differences in the abundance or phosphorylation status of Ca^2+^ cycling proteins in SANC and cells of working myocardium as well as impact of these varieties on cardiac pacemaking. The goal of this review is to discuss specific patterns of intrinsic SR Ca^2+^ cycling in SANC and ventricular myocytes, with the major focus on the SR Ca^2+^ cycling proteins (SERCA, PLB and RyR) expression, distribution and Ca^2+^-dependent modulation of the phosphorylation status of these proteins and functional implications with respect to generation of spontaneous LCRs and therefore regulation of spontaneous SANC beating rate.

## 2. Differences in Intrinsic SR Ca^2+^ Cycling between SANC and Ventricular Myocytes

The cardiac SR is designed to oscillate Ca^2+^ via Ca^2+^ pump, sarco-/endoplasmic reticulum Ca^2+^/ATPase (SERCA2), and RyRs. Electrical impulses, emanating from the SA node, reach ventricular myocardium and generate action potentials in cardiac myocytes, opening voltage gated L-type Ca^2+^ channels located in the t-tubular system. Ca^2+^ entering the cell through L-type Ca^2+^ channels open RyRs, which are normally packed in clusters near L-type Ca^2+^ channels to form Ca^2+^ release units (CRUs). Spontaneous SR-generated Ca^2+^ releases were originally discovered in ventricular myocytes and called “Ca^2+^ sparks”, which are relatively small in size ~2.0 μm and occur stochastically [59]. There are substantial species-dependent differences in Ca^2+^ handling, i.e., relaxation of cardiac myocytes occurs when Ca^2+^ is either taken back into the SR by SERCA2 or extruded from the cell by the sarcolemmal NCX. The contribution of each of these mechanisms is different among species, e.g., in the rabbit or human heart ~75% of Ca^2+^ is removed by SERCA2a and ~25% by NCX, while in the murine heart, including rats and mice, ~95% of Ca^2+^ is removed by SERCA2a [27]. Comparable amplitudes of I_NCX_ are observed in rabbit and human SANC when matched to the net diastolic current [60]. The differences in the contribution of NCX in Ca^2+^ fluxes of rabbit/human vs. mouse/rat heart could have a major impact on the modulation of cardiac pacemaking. Considering that the flux through NCX is ~4-fold less in mouse cells, an impact of I_NCX_ on spontaneous diastolic depolarization and consequently on mouse SANC firing rate is expected to be substantially less compared to the rabbit. 

All proteins, including SERCA, RyRs and NCX, that cycle Ca^2+^ in ventricular or atrial myocytes, are also present in SANC [19,21,61]. These proteins are found throughout the SA node, including the SA node center (connexin 43 deficient area) [61]. Similar to ventricular myocytes spontaneous Ca^2+^ oscillations occur in SANC but, compared to sparks in ventricular myocytes, local Ca^2+^ releases (LCRs) in SANC are relatively large, ~4–15 μm, and represent Ca^2+^ wavelets which likely involve several adjacent CRU [19,62].

A comparison of intracellular Ca^2+^ cycling in intact SANC and ventricular myocytes is hampered by the presence of different sets of membrane ion channels, which are also regulated by Ca^2+^ [1,2,3]. Cell surface membrane permeabilization is a valuable tool for studying intracellular Ca^2+^ cycling in the absence of ion channel interference in SANC [23,35] or ventricular myocytes [63,64,65,66]. This approach was employed to study mechanisms of CICR in cardiac cells [67]. In permeabilized cardiac cells, SR Ca^2+^ cycling becomes “free running”, controlled mostly by the concentration of free cytosolic Ca^2+^ [Ca^2+^]_f_ and the kinetics of Ca^2+^ pumping into and release from the SR. 

**Figure 2:**
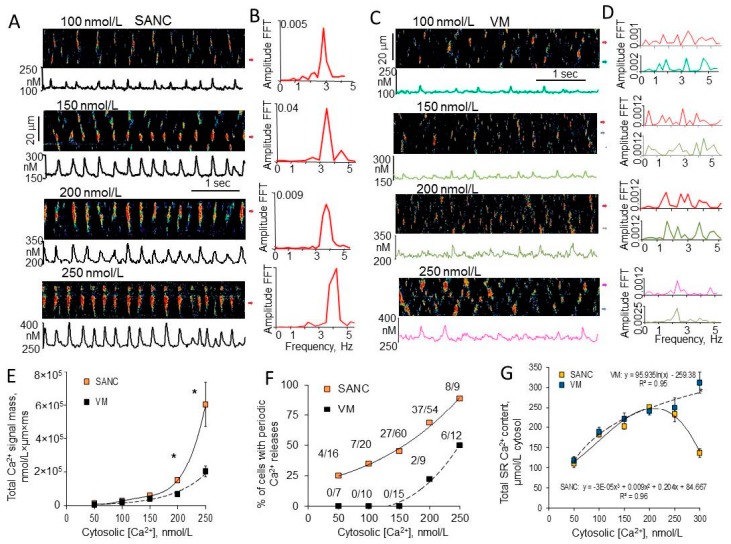
Spontaneous local Ca^2+^ releases are more robust and periodic in permeabilized rabbit SANC than in ventricular myocytes (VM). (**A**,**C**) Confocal images, Ca^2+^ waveforms; and (**B**,**D**) fast Fourier transform (FFT) of Ca^2+^ waveforms showed more rhythmic and large Ca^2+^ releases in saponin-permeabilized rabbit SANC than in ventricular myocytes bathed at the same cytosolic free [Ca^2+^]_f_ (modified from Sirenko et al. [35]). (**E**) Total amount of Ca^2+^ released from the SR (total Ca^2+^ signal mass) was comparable between SANC and ventricular myocytes at 50–100 nmol/L [Ca^2+^]_f_ and significantly higher in SANC than in ventricular myocytes at [Ca^2+^]_f_ > 150 nmol/L. (**F**) Relative number of cells generated periodic spontaneous Ca^2+^ releases was higher in SANC than that in ventricular myocytes at the same [Ca^2+^]_f_. (**G**) SR Ca^2+^ content was comparable in saponin-permeabilized SANC and VM at physiological Ca^2+^ concentration (50–250 nmol/L), and significantly higher in VM than in SANC at 300 nmol/L [Ca^2+^]_f_. *Indicates statistical significance (*p* < 0.05).

Under controlled physiological conditions and at the same level of free cytosolic [Ca^2+^]_f_ spontaneous LCRs in permeabilized SANC were large and roughly periodic events (Figure 2A,B). In contrast, sparks in permeabilized ventricular myocytes were relatively small and stochastic (Figure 2C,D). Total Ca^2+^ released by each cell type was estimated by integrating signal masses of all spontaneous LCRs in SANC and sparks in ventricular myocytes within a same time and space of the line-scan image [23]. At relatively low (50–100 nmol/L) cytosolic [Ca^2+^]_f_, both SANC and ventricular myocytes released comparable amount of Ca^2+^. However, even at this low cytosolic [Ca^2+^]_f_, almost 25–30% of SANC generated roughly periodic LCRs (periodicity was verified using fast Fourier transform (FFT)), while no periodic events were observed in ventricular myocytes up to 150 nmol/L [Ca^2+^]_f_. At higher 150–250 nmol/L cytosolic [Ca^2+^]_f_, the total Ca^2+^ signal mass released by SANC was ~2-fold larger compared to ventricular myocytes (Figure 2E). 

These concentrations of cytosolic [Ca^2+^]_f_ represent the most physiologically relevant range, since basal diastolic [Ca^2+^]_f_ concentration is ~160 nmol/L in spontaneously beating rabbit SANC [23] and ~250 nmol/L in intact paced ventricular myocytes [27,68]. Elevation of the cytosolic [Ca^2+^]_f_ increased number of cells with periodic Ca^2+^ releases in both cell types, but only ~20% of ventricular myocytes generated roughly periodic Ca^2+^ releases in ventricular myocytes at 200 nmol/L [Ca^2+^]_f_, while ~70% of SANC generated periodic LCRs. The differences in total Ca^2+^ signal mass and periodicity of Ca^2+^ releases between SANC or ventricular myocytes could be related to the variances in the SR Ca^2+^ load in two cell types. The global SR Ca^2+^ content in permeabilized SANC and ventricular myocytes, however, was the same over a wide range of physiologically relevant (50–250 nmol/L) cytosolic [Ca^2+^]_f_ concentrations (Figure 2G). Thus, SANCs sustain larger, rhythmic LCRs through RyRs at similar SR Ca^2+^ loads as ventricular myocytes. Further increase in cytosolic [Ca^2+^]_f_ to 300 nmol/L decreased the SR Ca^2+^ load and abolished spontaneous LCRs in SANC, while a further increase in the SR Ca^2+^ content and appearance of rhythmic Ca^2+^ wavelets was observed in ventricular myocytes [35]. Therefore, the rhythmicity of spontaneous Ca^2+^ events in both cell types was Ca^2+^-dependent, but SANC were more sensitive to [Ca^2+^]_f_ compared to ventricular myocytes. 

## 3. Differences in RyR Abundance and Distribution in SANC and Ventricular Myocytes

**Figure 3:**
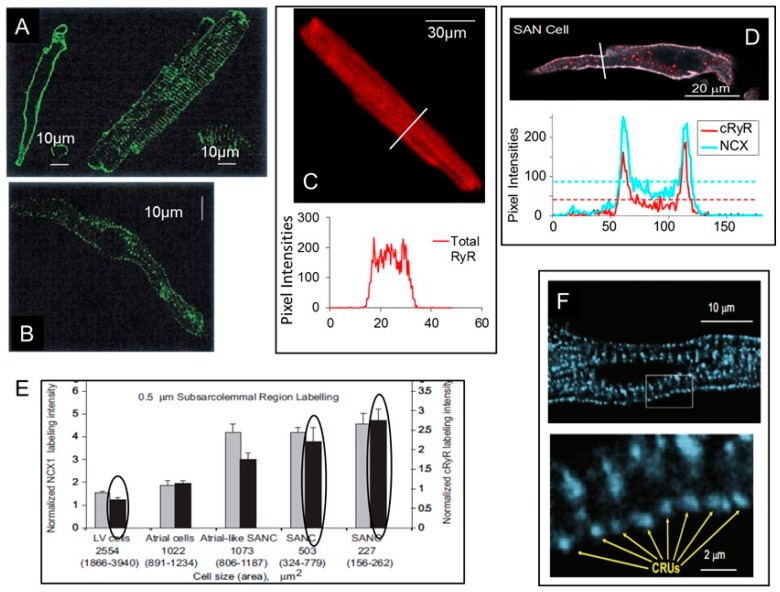
Distribution of ryanodine receptors (RyRs) and Na^+^/Ca^2+^ exchanger (NCX) in SANC. (**A**) The surface membrane labeled with di-8-ANEPPS in guinea-pig SANC (left panel) and VM (right panel) showed that SANCs, in contrast to VMs, have no invaginations associated with T-tubules (modified from Rigg et al. [21]). (**B**) Immunolabeling of guinea-pig SANC with anti-RyR antibody identified higher labeling intensity close to sarcolemma (modified from Rigg et al. [21]). (**C**) Immunostaining of rabbit saponin-permeabilized VM for total RyR (top) and the average pixel intensity (bottom) showed an even labeling intensity across the cell (modified from Sirenko et al. [35]). (**D**) An example of double immunolabeling of intact rabbit SANC with NCX and RyR antibodies (top); pixel-by-pixel intensities showed that NCX and RyR are colocalized in the submembrane regions of SANC (modified from Lyashkov et al. [61]). (**E**) Quantitative analysis of labeling intensities within a 0.5 µm subspace beneath the perimeter of the sarcolemma confirmed the significant increase in both NCX (grey bars) and RyR (black bars) in SANC compared to that in left ventricular (LV) myocytes (NCX1 labeling in SANCs differs from LV and right atrial cells by *p* < 0.05 post hocANOVA. cRyR labeling of SANCs differ from LV and right atrial cells, *p* < 0.01 post hoc ANOVA, modified from Lyashkov et al. [61]). (**F**) In rabbit SANC, immuno-fluorescence of RyR clusters, i.e., local Ca^2+^ release units (CRUs), showed their localization under the cell surface membrane with the average distance ~1.3 μm (modified from Maltsev et al. [69]).

Differences in spontaneous SR Ca^2+^ release characteristics in SANC and ventricular myocytes appear to be partially due to differences in the ultrastructure and abundance of proteins involved in the E-C coupling between two cell types. Specifically, T-tubular system is absent in SANC (Figure 3A), and RyR (especially in larger SANC) are distributed in the cytoplasm at regular bands localized at the Z-lines with periodicity of ~2 μm as well as beneath sarcolemma [21,61]. In small- and medium-sized SANCs, RyRs are mostly detected at the junctional SR (j-SR) in the proximity to sarcolemma (Figure 3B,D), while, in ventricular myocytes, RyRs are similarly present both at the cell surface and inside the cell (Figure 3C) [61]. Although the intensity of whole cell immunolabeling of RyR does not substantially vary between SANC and ventricular myocytes, quantitative analysis of RyR labeling intensities beneath sarcolemma established significant ~3-fold increase in RyR labeling density within a 0.5 μm subspace in SANC compared to left ventricular myocytes (Figure 3E) and in this area RyR were colocalized with NCX (Figure 3D). The fine structure of RyR in rabbit SANC was further studied using 2D-images, which showed irregularly spaced clusters of RyR of varied sizes (at least, at the light microscopy resolution). Some regions in subsarcolemmal area of SANC appeared to lack RyRs while other regions seemed to bridge the largest RyR clusters and link multiple CRUs in the network of RyR (Figure 3F) [62,69].

In isolated intact rabbit atrial myocytes (most of which also lack T-tubular system), simultaneous measurements of cytosolic [Ca^2+^] and SR Ca^2+^ depletions [Ca^2+^]_SR_ (fluo-5N) revealed that Ca^2+^ release from j-SR had larger Ca^2+^ spark amplitudes, but smaller SR Ca^2+^ depletions (Ca^2+^ blinks) compared to releases from the central non-junctional SR (nj-SR) [70], suggesting that higher cytosolic Ca^2+^ signal in the cell periphery could be due to a larger amount of Ca^2+^ released from the j-SR. Considering geometrical and structural factors, however, it has been hypothesized that in atrial myocytes j-SR release Ca^2+^ into the narrow cleft beneath sarcolemma. In this restricted cleft, cytosolic [Ca^2+^]_i_ might rapidly reach substantial levels. Since the same amount of Ca^2+^ released in the cell center is not confined by surrounding membranes, it could rapidly dissipate via Ca^2+^ buffering or repumping Ca^2+^ into SR and not reach comparable peak levels. Another hypothesis put forward in the same study was that there could be different pools of Ca^2+^ for j-SR and nj-SR or j-SR and nj-SR CRUs have different Ca^2+^ release termination mechanisms [70]. However, the relevance of these interesting hypotheses, created for atrial myocytes, to RyR Ca^2+^ release beneath sarcolemma of SANC requires further investigation.

## 4. Differences in SERCA Abundance among SANC, Atrial and Ventricular Myocytes

**Figure 4:**
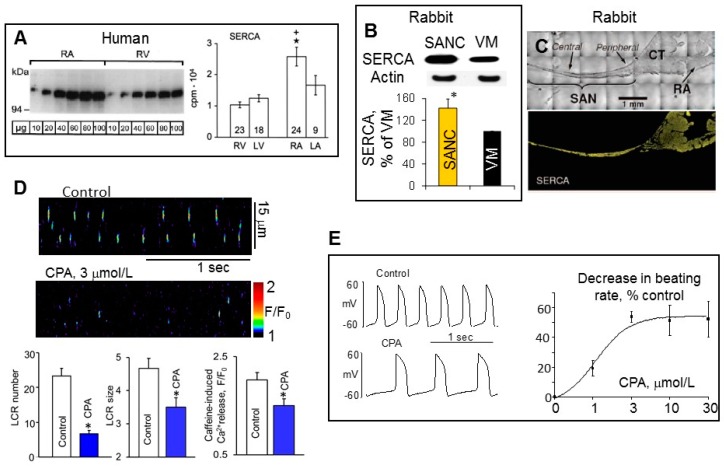
SR Ca^2+^ pump, SERCA, is more abundant in SANC than in ventricular myocytes. (**A**) Representative Western blot (left) and average data (right) showed that SERCA is more abundant in the human right atrium (RA) compared to right (RV) or left (LV) ventricle (* *p* < 0.05 vs. RV, + *p* < 0.05 vs. LV, Bonferroni post hoc ANOVA; modified from Boknık et al. [75]). (**B**) Representative Western blots (top) and average data (bottom) confirmed increased abundance of SERCA in rabbit SANC compared to ventricular myocytes (VM) (modified from Sirenko et al. [35]). (**C**) Immunolabeling of rabbit SA node tissue sections showed robust SERCA labeling within the center and periphery of the SA node (modified from Lyashkov et al. [61]). (**D**) Suppression of SERCA by cyclopiazonoc acid (CPA, 3 µmol/L) significantly decreased average LCR number, size and SR Ca^2+^ load in permeabilized rabbit SANC (modified from Vinogradova et al. [36]). (**E**) CPA dose-dependently decreased spontaneous firing rate of intact rabbit SANC (modified from Vinogradova et al. [36]). * Indicates statistical significance (*p* < 0.05).

In permeabilized cells, in the absence of functional sarcolemmal ionic channels, but intact function of SR, the SR Ca^2+^ content is largely regulated by the release of Ca^2+^ through RyR and Ca^2+^ pumping into SR by SERCA. Ca^2+^ pumping by SERCA is a determinant of the decay of the intracellular Ca^2+^ transient and refilling of the SR Ca^2+^ content [71]. SERCA2 is a 110 kD transmembrane SR protein expressed in all cardiac myocytes, which maintains a 1000-fold Ca^2+^-gradient across the cardiac SR membrane [72]. In multiple species including rabbit, guinea pig and human (Figure 4A), SERCA2a protein level is approximately two-fold higher in atria compared to ventricle [73], which may partly account for the shorter duration of contraction in atrial vs. ventricular tissue [74]. 

In ventricular myocytes the total RyR Ca^2+^ release controls the amount of Ca^2+^ stored in the SR and therefore provides powerful negative feedback regulation. For example, if RyR Ca^2+^ release is acutely elevated, Ca^2+^ efflux from the cell also increases, reducing the amount of Ca^2+^ available for pumping into SR, limiting the SR Ca^2+^ content and thus decreasing subsequent RyR Ca^2+^ releases [76]. The total Ca^2+^ signal mass released by SANC is ~2-fold larger compared to ventricular myocytes when cytosolic [Ca^2+^]_f_ is 150–250 nmol/L (Figure 2E). Elevated RyR Ca^2+^ release in SANC, however, produced no detectable depletion of SR Ca^2+^ content (Figure 2G), strongly suggesting that Ca^2+^ pumping into SR by SERCA was higher in SANC than in ventricular myocytes. Western blots of SERCA in isolated SANC and ventricular myocytes demonstrated that the abundance of SERCA in rabbit SANC exceeded that in ventricular myocytes by ~1.5-fold (Figure 4B), and SERCA was uniformly distributed within rabbit SANC [61]. Both the central SA node area (connexin 43 negative area) and SA node periphery exhibited positive immunoreactivity for SERCA2 (Figure 4C) [61]. 

The direct effect of SERCA2 pumping on intrinsic SR Ca^2+^ cycling in permeabilized SANC was verified employing specific and reversible SERCA inhibitor cyclopiazonic acid [77], which markedly reduced the LCR frequency and size in permeabilized SANCs partially due to a substantial reduction in the SR Ca^2+^ content (Figure 4D). In intact rabbit SANC, cyclopiazonic acid decreased the spontaneous SANC beating rate in a dose-dependent manner (EC_50_, 1.2 μmol/L) to a maximal suppression of ~50% (Figure 4E), and all effects were reversed after washout. The suppression in the SANC beating rate was attributable to a marked decrease in the diastolic depolarization rate, indicating that SR refilling plays an essential role in the regulation of the basal cardiac pacemaker function. 

The effects of variations in levels of SERCA2a was studied by either SERCA2a overexpression or deletion in mouse models. Transgenic mice overexpressing SERCA2a protein showed no cardiac pathology, but exhibited augmented SR Ca^2+^ transport with enhanced rates of cardiac contractility and relaxation [78]. Specifically, an increase in SERCA2a protein levels by ~1.5-fold in transgenic mice was associated with elevation of the maximum velocity of SR Ca^2+^ uptake by ~40%, demonstrating that increased pump level results in increased SR Ca^2+^ uptake function. Hearts from SERCA2a transgenic mice showed significantly higher myocardial contractile function and slightly increased spontaneous beating rate [79], indicating that SERCA is a key determinant of both cardiac contraction and spontaneous SANC firing. Absence of the SERCA2 gene is embryonically lethal and homozygous (SERCA2 −/−) mice die in development. In adult mice, an inducible cardiomyocyte-specific excision of the Atp2a2 (Serca2) gene (SERCA2 KO) produced dramatic reduction in the SR Ca^2+^ content which reached only ~20% of control values in SERCA2 KO cardiomyocytes [80,81]. The heart rate was also significantly lower in anesthetized SERCA2 KO mice, which could be due to deletion of SERCA2 in the sinus node [80].

There is a decrease in the expression levels of SERCA2a and its activity with ageing both in animal models and in senescent human myocardium [82,83,84]. The decrease in SERCA2 protein in the SA node isolated from aged (20–24 months) vs. adult (3–4 months) mice was linked to decrease in SR Ca^2+^ load, resultant reduction in LCR size and number and age-associated decrease in the spontaneous SANC beating rate [85]. In failing human hearts, SERCA2a function is impaired due to reduction in SERCA2a mRNA and protein levels [86,87,88,89,90,91]. Restoration of normal levels of SERCA2a has been suggested as a novel therapeutic target for treatment of the heart failure [92,93,94,95].

## 5. Differences in Phospholamban (PLB) Abundance in SANC and Ventricular Myocytes

The activity of SERCA2a is modulated by multiple factors, the predominant factor being a 52 amino-acid phosphoprotein PLB, which, in its unphosphorylated form, colocalizes with SERCA2a in the cardiac SR membrane [96] and inhibits its function [97]. Since PLB is an inhibitor of SERCA pump, an increase or decrease in PLB level can directly impact SR Ca^2+^ uptake function. For example, the reduction in PLB levels in ventricle was associated with a linear increase in the affinity of SERCA2a for Ca^2+^, and resultant increase of contractility [97,98]. 

**Figure 5:**
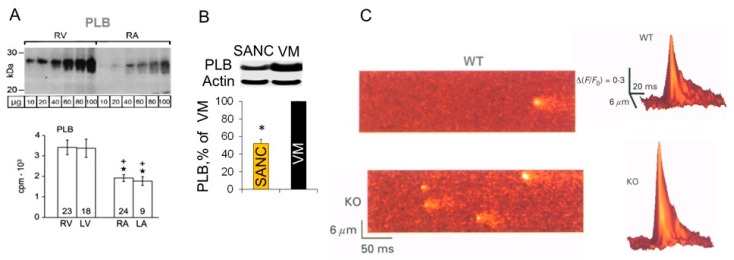
Comparison PLB abundance in SANC and ventricular myocytes. (**A**) Representative Western blot (top) and average data (bottom) showed that PLB is less expressed in human right (RA) or left (LA) atrium than in right (RV) or left (LV) ventricle (* *p* < 0.05 vs. RV, + *p* < 0.05 vs. LV, Bonferroni post hoc ANOVA; modified from Boknık et al. [75]). (**B**) Representative Western blot (top) and average data (bottom) showed that PLB was less abundant in rabbit SANC than in VM (modified from Sirenko et al. [35]). (**C**) Confocal line-scan images (left) and surface plots (right) of WT and PLB-KO mouse VM showed that spontaneous Ca^2+^ sparks were three times more frequent and larger in amplitude in KO cells than that in WT (modified from Santana et al. [99]]). * Indicates statistical significance (*p* < 0.05).

Compared to smooth muscle tissue, PLB is expressed at higher levels in the heart, but expression of PLB protein in many species, including humans (Figure 5A), rabbits, guinea pigs, mice and rats, is ~2–3-fold higher in ventricle compared to atria [73,75]. The abundance of PLB in rabbit SANC was ~2-fold less compared to ventricular myocytes, indicating that inhibition of SERCA by PLB could be lower in SANC than in ventricular myocytes (Figure 5B). 

On the other hand, SERCA protein was ~1.5-fold more abundant in SANC compared to ventricular myocytes (Figure 4B). Thus, the SERCA/PLB ratio could be at least ~3-fold larger in SANC than in ventricular myocytes, suggesting that the difference in Ca^2+^ cycling proteins abundance partially accounts for the differences in SR Ca^2+^ releases between SANC and ventricular myocytes. Indeed, robust local Ca^2+^ releases in SANC are supported by more activated (uninhibited) SERCA, which could provide an increased Ca^2+^ pumping into the SR required to sustain long-lasting elevated RyR Ca^2+^ releases. The PLB to SERCA ratio considered to be a key factor of SR Ca^2+^ cycling and cardiac contractility regulation [100]. In theory, a lower level of PLB could facilitate rate of SR Ca^2+^ uptake, elevating the SR Ca^2+^ content and providing more Ca^2+^ for SR to release. Conversely, an increase in PLB level would decrease rate of SR Ca^2+^ uptake, reduce the SR Ca^2+^ load and, as a result, suppress the SR Ca^2+^ release. Indeed, atrial muscles, which have a ~3–4-fold lower PLB to SERCA2 ratio than ventricular muscles, exhibited rates of force development and relaxation of tension, which were ~3-fold faster compared to ventricular muscles [100,101]. 

The inhibitory role of PLB on SR and cardiac function has been directly confirmed using transgenic mouse models. In ventricular myocytes isolated from PLB KO mice [Ca^2+^]_i_ transient was larger and decayed faster compared to WT mice [99,102]. Spontaneous Ca^2+^ sparks were also markedly increased in size and were ~3 times more frequent in ventricular myocytes from PLB KO mice compared to WT mice (Figure 5C), which was partially due to the elevated SR Ca^2+^ load [99]. The adenoviral gene transfer of antisense PLB in human cardiomyocytes isolated from failing hearts decreased expression of PLB, restoring the velocity of both contraction and relaxation in the failing cardiomyocytes to normal [103]. Although a lack of PLB in the mouse heart is well tolerated, a similar condition in human leads to dilated cardiomyopathy and death [104,105]. 

## 6. High Basal PKA- and CaMKII-Dependent Phosphorylation in Cardiac Pacemaker Cells

**Figure 6:**
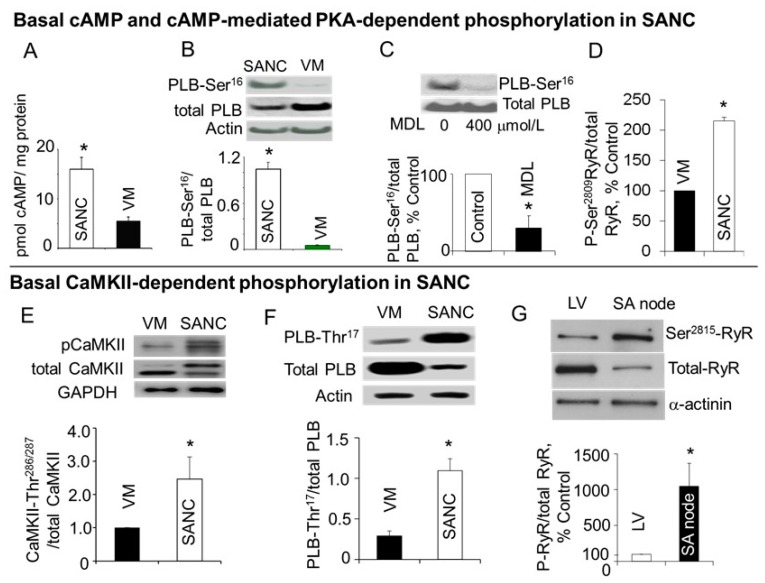
Basal levels of cAMP, PKA- and CaMKII-dependent phosphorylation in rabbit SANC and ventricular myocytes (VM). (**A**) Basal level of cAMP was substantially higher in SANC than in ventricular myocytes (modified from Vinogradova et al. [113]). (**B**) Basal level of PLB phosphorylation was significantly increased at PKA-dependent Ser^16^ site in SANC compared to VM (modified from Vinogradova et al. [106]). (**C**) Adenylyl cyclase inhibitor, MDL-12,330A, suppressed PLB phosphorylation at Ser^16^ site in SANC (modified from Vinogradova et al. [106]). (**D**) Basal level of RyR phosphorylation at Ser^2809^ site was markedly higher in SANC compared to VM (modified from Li et al. [39]). (**E**) Basal level of activated (autophosphorylated at Thr^286/287^) CaMKII (pCaMKII) was ~2-fold higher in SANC compared to ventricular myocytes (modified from Li et al. [39]). (**F**) Basal phosphorylation of PLB at the CaMKII-dependent Thr^17^ site (PLB-Thr^17^) was ~3-fold higher in SANC compared to VM (modified from Li et al. [39]). (**G**) Basal phosphorylation of RyR at CaMKII-dependent Ser^2815^ site (Ser^2815^-RyR) was ~10-fold higher in the rabbit SA node than that in the left ventricle (LV) (modified from Li et al. [39]]). * Indicates statistical significance (*p* < 0.05).

Another reason why LCRs in rabbit SANC are more robust compared to ventricular myocytes (Figure 2) might be that, even in the absence of β-AR stimulation, basal cAMP level in SANC is ~3-fold higher compared to ventricular myocytes (Figure 6A). The elevated level of cAMP in the basal state is due to constitutive activation of adenylyl cyclases (AC), since AC inhibitor, MDL-12,330A, markedly suppresses both basal level of cAMP and spontaneous beating rate of rabbit SANC [106]. The constitutive AC activation, however, is not triggered by constitutive β-AR activation, since neither the β1-AR antagonist, CGP-20712A, nor the β2-AR subtype inverse agonist, ICI 118,551, affects the spontaneous SANC beating rate [106]. The predominant AC isoforms in the ventricle are Ca^2+^-inhibited AC5 and AC6 [107,108,109]. Similar to ventricular myocytes, SANCs also express AC5/6; however, they also express Ca^2+^-activated AC1 and AC8, which are distributed close to sarcolemma [110] and appear to reside largely within caveolin-enriched membrane microdomains [111]. 

Basal protein kinase A (PKA)- and Ca^2+^/calmodulin-dependent protein kinase II (CaMKII)-dependent protein phosphorylation is required to sustain normal spontaneous firing of cardiac pacemaker cells. When basal PKA- or CaMKII-dependent phosphorylation is inhibited, spontaneous beating of SANC is reduced and ultimately ceased [28,39,106,112]. The intrinsic “Ca^2+^ clock” of rabbit SANC is also regulated by basal activation of both PKA- and CaMKII-dependent protein phosphorylation (Figure 1C), which determines the amount of Ca^2+^ available for pumping and modulates the speed of Ca^2+^ pumping into SR and its release through activation of RyRs by internal SR Ca^2+^ and RyR phosphorylation. 

It is well established that phosphorylation of PLB by PKA or CaMKII in ventricular myocytes releases inhibition of SERCA and elevates SERCA activity by ~2–3-fold [97,114,115,116,117,118,119]. SERCA activation speeds up re-uptake of Ca^2+^ into SR, shortens duration of the [Ca^2+^]_i_ transient and reduces duration of Ca^2+^ sparks [120]. Basal level of PLB phosphorylation at the PKA-dependent Ser^16^ site was substantially higher in SANC compared to ventricular myocytes (Figure 6B), and inhibition of either AC activity (Figure 6C) or PKA-dependent phosphorylation markedly decreased basal PLB phosphorylation [106], indicating that functionally relevant high basal PKA activation was driven by a high basal activity of AC. There was a close correlation between gradations in basal PKA activity, indexed by changes in PLB phosphorylation and altered by selective PKA inhibitory peptide PKI, and concomitant suppression of LCR characteristics and spontaneous SANC beating rate [106]. Basal level of RyR phosphorylation at Ser^2809^ site was also ~2-fold higher in isolated rabbit SANC compared with VM (Figure 6D), and inhibition of either PKA-dependent phosphorylation by PKI or CaMKII-dependent phosphorylation by KN-93 suppressed RyR phosphorylation at this site, indicating that this site in SANC, as in VM, is phosphorylated by both protein kinases [39]. 

The basal level of activated (autophosphorylated) CaMKII in rabbit SANC exceeded that in ventricular myocytes by ~2-fold (Figure 6E) and this was accompanied by high basal phosphorylation of PLB at CaMKII-dependent Thr^17^ site, which was ~3-fold greater in SANC compared to ventricular myocytes (Figure 6F) [39]. Total CaMKII is uniformly distributed within rabbit SANC, activated (autophosphorylated) CaMKII, however, is localized beneath sarcolemma, supporting the idea that CaMKII targets sarcolemmal and subsarcolemmal compartments, and that CaMKII activity, in its turn, is likely regulated by variations in local Ca^2+^ beneath sarcolemma [112,121]. 

In cardiac myocytes, CaMKII is directly associated with RyR [122,123,124], and CaMKIIδ-dependent RyR phosphorylation increases SR Ca^2+^ leak and Ca^2+^ sparks in cardiac myocytes [122] increasing Ca^2+^ sensitivity and open probability of RyR [123,124,125,126]. Basal RyR phosphorylation at CaMKII-dependent Ser^2815^ site (Figure 6G) was ~10-fold higher in the SA node compared to ventricle [39], which could be partly explained by similar distribution of both RyR [61] and activated CaMKII beneath sarcolemma of rabbit SANC [112].

L-type Ca^2+^ channels are part of both “Membrane clock” and “Ca^2+^ clock”, since they generate action potential upstroke in primary cardiac pacemaker cells and provide Ca^2+^ supply for pumping into SR. There is a high basal PKA- and CaMKII-dependent phosphorylation of L-type Ca^2+^ channels in rabbit SANC, as a specific PKA inhibitor peptide, PKI, or CaMKII inhibitors, KN-93 or AIP, suppressed I_CaL_ by ~80% [127] and ~50% [112], respectively. 

Basal CaMKII-dependent phosphorylation has been implicated in the regulation of basal SR Ca^2+^ cycling and spontaneous beating of rabbit SANC [39,112]. Recent studies in transgenic mice [128,129,130,131], however, did not find any differences in the basal heart beating rate between wild type mice and mice with either conditional inhibition of CaMKII (AC3-I mice) [31,131], or CaMKIIδ knockout (KO) mice [130]. Moreover, in contrast to rabbit SANC, there was no basal CaMKII-dependent phosphorylation in mouse SANC isolated either from the wild type or CaMKII KO mice [31]. Absence of basal CaMKII-dependent phosphorylation in mouse SANC may explain, why normal cardiac pacemaker function was preserved in transgenic mice with conditional CaMKII inhibition [31,130]. It is likely that CaMKII-dependent modulation of SR Ca^2+^ cycling could be redundant, which is also consistent with a less important contribution of I_NCX_ in the regulation of cardiac pacemaker function in mouse SANC. 

## 7. Ca^2+^-Dependent Phosphorylation of Both PLB and RyRs Was Associated with Amplified Ca^2+^ Release from SR

**Figure 7:**
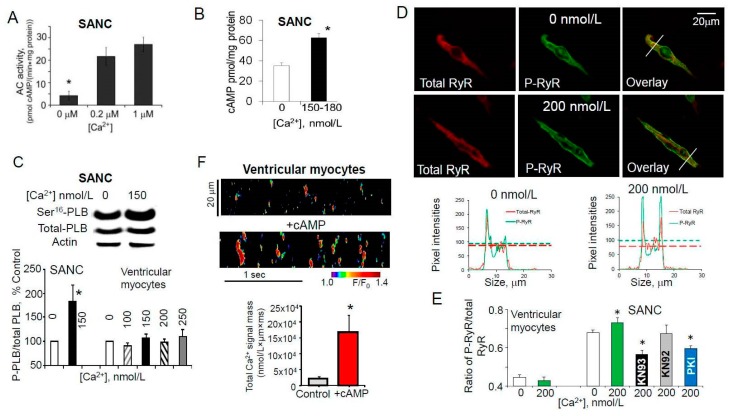
Ca^2+^-dependent modulation of cAMP and PKA-dependent phosphorylation in rabbit SANC, but not in ventricular myocytes. (**A**) Ca^2+^-dependent increase of AC activity of whole cell lysates of intact SANC (modified from Younes et al. [9]). (**B**–**E**) (modified from Sirenko et al. [35]): (**B**) An increase in cytosolic free [Ca^2+^]_f_ produced an increase in cAMP concentration in permeabilized SANC. (**C**) An increase in cytosolic free [Ca^2+^]_f_ increased phosphorylation of PLB at Ser^16^ site in permeabilized SANC, but not in ventricular myocytes. (**D**) Representative permeabilized SANC immunolabeled for total and phosphorylated RyR at Ser^2809^ (kept at 0 (top) or 200 nmol/L (middle) [Ca^2+^]_f_ respectively); pixel-by-pixel fluorescence intensities (bottom) showed that increase in cytosolic [Ca^2+^]_f_ produced an increase in RyR phosphorylation. (**E**) Average data of changes in RyR phosphorylation at Ser^2809^ produced by an increase in cytosolic [Ca^2+^]_f_ in permeabilized SANC and ventricular myocytes. Ca^2+^-stimulated RyR phosphorylation in SANC at 200 nmol/L [Ca^2+^]_f_ could be partially reversed by inhibition of PKA by PKA inhibitor peptide (PKI) or CaMKII inhibitor (KN-93, but not its inactive analog KN-92), indicating that this site is phosphorylated by both PKA and CaMKII. (**F**) An increase in cAMP level in permeabilized rabbit ventricular myocytes transformed stochastic Ca^2+^ sparks into more rhythmic and large Ca^2+^ wavelets (top and middle panels) and significantly increased total amount of Ca^2+^ released from the SR (total Ca^2+^ signal mass, bottom panel) (modified from Sirenko et al. [66]). * Indicates statistical significance (*p* < 0.05).

As noted above, in addition to non-Ca^2+^-activated AC isoforms rabbit SANC express two Ca^2+^-activated isoforms AC1 and AC8, and the main part of basal AC activity in rabbit SANC is Ca^2+^-activated (Figure 7A) [110,111,132]. Consistent with this idea, an increase in the cytosolic [Ca^2+^]_f_ concentration in permeabilized SANC was associated with an increased level of cAMP (Figure 7B), indicating activation of ACs and, as a result, augmentation of cAMP production. As expected, an increase in cAMP level in permeabilized SANC was accompanied by in an increase in PLB phosphorylation at Ser^16^ (Figure 7C), which should relieve SERCA inhibition and increase pumping Ca^2+^ into SR. Increase in physiological cytosolic [Ca^2+^]_f_ in permeabilized SANC would likely activate CaMKII [133,134], and, indeed, there was a marked increase in CaMKII-dependent phosphorylation of both PLB [35] and RyR at Ser^2809^ site (Figure 7D,E), which is phosphorylated by both PKA and CaMKII in rabbit SANC [35]. Thus, coordinated and synchronized Ca^2+^-dependent increase in both Ca^2+^ release and reuptake into SR could sustain large and rhythmic spontaneous LCRs in rabbit SANC and maintain sufficient SR Ca^2+^ load (Figure 2G) in the presence of an augmented RyR Ca^2+^ release. 

In contrast, an increase in the cytosolic [Ca^2+^]_f_ concentration in permeabilized ventricular myocytes produced no changes in PLB phosphorylation either at Ser^16^ (Figure 7C) or Thr^17^ sites [35], suggesting different Ca^2+^-dependent regulation of PKA- and CaMKII-dependent phosphorylation in these two cell types. Ca^2+^-inhibited AC5/6 are the most abundant AC isoforms in the ventricle [135], and that might explain why an elevation of cytosolic [Ca^2+^]_f_ in permeabilized ventricular myocytes produced no increase in PKA-dependent phosphorylation of either PLB or RyR. Ca^2+^ sparks in ventricular myocytes are relatively small and stochastic which could be partially due to a low level of basal SR Ca^2+^ cycling protein phosphorylation. An increase in phosphorylation status of SR Ca^2+^ cycling proteins (PLB and RyR) by addition of exogenous cAMP (Figure 7F) or inhibition of protein phosphatases or phosphodiesterases changed stochastic Ca^2+^ sparks in permeabilized rabbit ventricular myocytes into periodic Ca^2+^ wavelets that resembled LCRs in SANC [66]. A similar effect was observed during addition of antibody (2D12) [136,137], that mimics the effect of PLB phosphorylation by inhibiting the PLB-SERCA2 interaction and increasing Ca^2+^ pumping into SR without directly effecting the PLB phosphorylation [66]. Therefore, a Ca^2+^ clock is not exclusive to pacemaker cells but can also be unleashed in ventricular myocytes when SR Ca^2+^ cycling is stimulated and spontaneous local Ca^2+^ releases become partially synchronized. 

The interplay between SR Ca^2+^ release via RyRs and reuptake by SERCA is likely to be a major factor that regulates LCR periodicity in rabbit SANC. Indeed, each local Ca^2+^ release produces local depletion of SR Ca^2+^ content, and the next spontaneous LCR occur after SR Ca^2+^ content is replenished by SERCA. Robust, rhythmic LCRs in SANC require high basal PKA- and CaMKII-dependent protein phosphorylation as inhibition of either PKA- or CaMKII-dependent phosphorylation resulted in small stochastic Ca^2+^ releases that resembled Ca^2+^ sparks in ventricular myocytes [35]. 

## 8. Conclusions

Our understanding of cardiac automaticity has progressed considerably and now includes not only electrophysiological description of ionic channels, i.e., “Membrane clock” in SANC, but also the intracellular SR Ca^2+^ cycling, i.e., “Ca^2+^ clock”, as well as interactions between these clocks within coupled-clock system. Intracellular SR Ca^2+^ cycling has distinct functions in pacemaker and cardiac muscle cells: in the former, it is involved in the generation of spontaneous action potentials, which are conducted to ventricular myocytes, and through excitation–contraction coupling initiate heartbeats. This has led to a general theory of cardiac chronotropy and inotropy [28]. Studies of the differences in the protein abundance between SA node and working myocardium as well as functional implications of these variances have recently emerged. In this review, we have characterized and compared differences in the abundance of Ca^2+^ handling proteins, including SERCA, PLB and RyR, between rabbit SANC and ventricular myocytes. Although there is overwhelming evidence that alterations in the ratio of SERCA to PLB can markedly change the SERCA pump activity and affect function of cardiac muscle cells, these ideas are relatively new to the field of cardiac pacemaker mechanisms. Our review emphasizes an increased abundance of SERCA in conjunction with reduced abundance of PLB protein in SANC compared to ventricular myocytes. A unique concurrent Ca^2+^-dependent regulation of both PKA- and CaMKII-dependent protein phosphorylation in rabbit SANC further accentuates differences between two cell types and links Ca^2+^ handling protein abundance and phosphorylation to the functional effects on intracellular SR Ca^2+^ cycling and cardiac pacemaker function.

Many important and complex questions, however, remain to be answered: Is the species-specific differences in cardiac pacemaker beating rate (~70 bpm in humans vs. ~400–800 bpm in mice) associated with the expression of proteins comprising ion channels or proteins regulating Ca^2+^ handling, or both? Why is loss of PLB beneficial in the mouse heart, but harmful in human? Why is basal CaMKII-dependent phosphorylation of Ca^2+^ cycling proteins augmented in rabbit SANC, but is apparently absent in the mouse SANC? Further studies of specific protein expression in SANC of different species as well as phosphorylation-dependent modulation of these proteins are required to uncover more complex physiological and pathological mechanisms that govern the heart pacemaker function.

## References

[1] Baruscotti M., Robinson R.B. (2007). Electrophysiology and pacemaker function of the developing sinoatrial node. Am. J. Physiol. Heart Circ. Physiol..

[2] Irisawa H., Brown H.F., Giles W. (1993). Cardiac Pacemaking in the Sinoatrial Node. Physiol. Rev..

[3] Mangoni M.E., Nargeot J. (2008). Genesis and regulation of the heart automaticity. Physiol. Rev..

[4] Monfredi O., Dobrzynski H., Mondal T., Boyett M.R., Morris G.M. (2010). The anatomy and physiology of the sinoatrial node--a contemporary review. Pacing Clin. Electrophysiol..

[5] Cranefield P. (1975). The Conduction of the Cardiac Impulse: The Slow Response and Cardiac Arrythmias.

[6] Seifen E., Schaer H., Marshall J.M. (1964). Effect of Calcium on the Membrane Potentials of Single Pacemaker Fibres and Atrial Fibres in Isolated Rabbits Atria. Nature.

[7] Hagiwara N., Irisawa H. (1989). Modulation by intracellular Ca^2+^ of the hyperpolarization-activated inward current in rabbit single sino-atrial node cells. J. Physiol..

[8] Hagiwara N., Irisawa H., Kameyama M. (1988). Contribution of two types of calcium currents to the pacemaker potentials of rabbit sino-atrial node cells. J. Physiol..

[9] Heath B.M., Terrar D.A. (2000). Protein kinase C enhances the rapidly activating delayed rectifier potassium current, IKr, through a reduction in C-type inactivation in guinea-pig ventricular myocytes. J. Physiol..

[10] Tohse N. (1990). Calcium-sensitive delayed rectifier potassium current in guinea pig ventricular cells. Am. J. Physiol..

[11] Guo J.Q., Ono K.I., Noma A.N. (1995). A Sustained Inward Current Activated at the Diastolic Potential Range in Rabbit Sinoatrial Node Cells. J. Physiol..

[12] Hata T., Noda T., Nishimura M., Watanabe Y. (1996). The role of Ca^2+^ release from sarcoplasmic reticulum in the regulation of sinoatrial node automaticity. Heart Vessel..

[13] Huser J., Blatter L.A., Lipsius S.L. (2000). Intracellular Ca^2+^ release contributes to automaticity in cat atrial pacemaker cells. J. Physiol..

[14] Ju Y.K., Allen D.G. (1998). Intracellular calcium and Na^+^-Ca^2+^ exchange current in isolated toad pacemaker cells. J. Physiol..

[15] Li J., Qu J., Nathan R.D. (1997). Ionic basis of ryanodine’s negative chronotropic effect on pacemaker cells isolated from the sinoatrial node. Am. J. Physiol..

[16] Rigg L., Terrar D.A. (1996). Possible role of calcium release from the sarcoplasmic reticulum in pacemaking in guinea-pig sino-atrial node. Exp. Physiol..

[17] Rubenstein D.S., Lipsius S.L. (1989). Mechanisms of automaticity in subsidiary pacemakers from cat right atrium. Circ. Res..

[18] Satoh H. (1997). Electrophysiological actions of ryanodine on single rabbit sinoatrial nodal cells. Gen. Pharmacol..

[19] Bogdanov K.Y., Vinogradova T.M., Lakatta E.G. (2001). Sinoatrial nodal cell ryanodine receptor and Na(^+^)-Ca(^2+^) exchanger: Molecular partners in pacemaker regulation. Circ. Res..

[20] Lipsius S.L., Huser J., Blatter L.A. (2001). Intracellular Ca^2+^ release sparks atrial pacemaker activity. Physiology.

[21] Rigg L., Heath B.M., Cui Y., Terrar D.A. (2000). Localisation and functional significance of ryanodine receptors during beta-adrenoceptor stimulation in the guinea-pig sino-atrial node. Cardiovasc. Res..

[22] Sanders L., Rakovic S., Lowe M., Mattick P.A., Terrar D.A. (2006). Fundamental importance of Na^+^-Ca^2+^ exchange for the pacemaking mechanism in guinea-pig sino-atrial node. J. Physiol..

[23] Vinogradova T.M., Zhou Y.Y., Maltsev V., Lyashkov A., Stern M., Lakatta E.G. (2004). Rhythmic ryanodine receptor Ca^2+^ releases during diastolic depolarization of sinoatrial pacemaker cells do not require membrane depolarization. Circ. Res..

[24] Maltsev V.A., Vinogradova T.M., Lakatta E.G. (2006). The emergence of a general theory of the initiation and strength of the heartbeat. J. Pharmacol. Sci..

[25] Lakatta E.G., Vinogradova T., Lyashkov A., Sirenko S., Zhu W., Ruknudin A., Maltsev V.A. (2006). The integration of spontaneous intracellular Ca^2+^ cycling and surface membrane ion channel activation entrains normal automaticity in cells of the heart’s pacemaker. Ann. N. Y. Acad. Sci..

[26] Maltsev V.A., Lakatta E.G. (2009). Synergism of coupled subsarcolemmal Ca^2+^ clocks and sarcolemmal voltage clocks confers robust and flexible pacemaker function in a novel pacemaker cell model. Am. J. Physiol. Heart Circ. Physiol..

[27] Bers D.M. (2001). Excitation-Contraction Coupling and Cardiac Contractile Force.

[28] Lakatta E.G., Maltsev V.A., Vinogradova T.M. (2010). A coupled SYSTEM of intracellular Ca^2+^ clocks and surface membrane voltage clocks controls the timekeeping mechanism of the heart’s pacemaker. Circ. Res..

[29] Joung B., Tang L., Maruyama M., Han S., Chen Z., Stucky M., Jones L.R., Fishbein M.C., Weiss J.N., Chen P.S. (2009). Intracellular calcium dynamics and acceleration of sinus rhythm by beta-adrenergic stimulation. Circulation.

[30] Sirenko S.G., Yang D., Maltseva L.A., Kim M.S., Lakatta E.G., Maltsev V.A. (2017). Spontaneous, local diastolic subsarcolemmal calcium releases in single, isolated guinea-pig sinoatrial nodal cells. PLoS ONE.

[31] Wu Y.J., Gao Z., Chen B.Y., Koval O.M., Singh M.V., Guan X.Q., Hund T.J., Kutschke W., Sarma S., Grumbach I.M. (2009). Calmodulin kinase II is required for fight or flight sinoatrial node physiology. Proc. Natl. Acad. Sci. USA.

[32] Tsutsui K., Monfredi O.J., Sirenko-Tagirova S.G., Maltseva L.A., Bychkov R., Kim M.S., Ziman B.D., Tarasov K.V., Tarasova Y.S., Zhang J. (2018). A coupled-clock system drives the automaticity of human sinoatrial nodal pacemaker cells. Sci. Signal..

[33] Choate J.K., Feldman R. (2003). Neuronal control of heart rate in isolated mouse atria. Am. J. Physiol. Heart Circ. Physiol..

[34] Meissner G. (1986). Ryanodine activation and inhibition of the Ca^2+^ release channel of sarcoplasmic reticulum. J. Biol. Chem..

[35] Sirenko S., Yang D., Li Y., Lyashkov A.E., Lukyanenko Y.O., Lakatta E.G., Vinogradova T.M. (2013). Ca^2+^-dependent phosphorylation of Ca^2+^ cycling proteins generates robust rhythmic local Ca^2+^ releases in cardiac pacemaker cells. Sci. Signal..

[36] Vinogradova T.M., Brochet D.X., Sirenko S., Li Y., Spurgeon H., Lakatta E.G. (2010). Sarcoplasmic reticulum Ca^2+^ pumping kinetics regulates timing of local Ca^2+^ releases and spontaneous beating rate of rabbit sinoatrial node pacemaker cells. Circ. Res..

[37] Lyashkov A.E., Beahr J., Lakatta E.G., Yaniv Y., Maltsev V.A. (2018). Positive Feedback Mechanisms among Local Ca Releases, NCX, and I-CaL Ignite Pacemaker Action Potentials. Biophys. J..

[38] Yaniv Y., Sirenko S., Ziman B.D., Spurgeon H.A., Maltsev V.A., Lakatta E.G. (2013). New evidence for coupled clock regulation of the normal automaticity of sinoatrial nodal pacemaker cells: Bradycardic effects of ivabradine are linked to suppression of intracellular Ca^2+^ cycling. J. Mol. Cell. Cardiol..

[39] Li Y., Sirenko S., Riordon D.R., Yang D., Spurgeon H., Lakatta E.G., Vinogradova T.M. (2016). CaMKII-dependent phosphorylation regulates basal cardiac pacemaker function via modulation of local Ca^2+^ releases. Am. J. Physiol. Heart Circ. Physiol..

[40] Yavari A., Bellahcene M., Bucchi A., Sirenko S., Pinter K., Herring N., Jung J.J., Tarasov K.V., Sharpe E.J., Wolfien M. (2017). Mammalian gamma 2 AMPK regulates intrinsic heart rate. Nat. Commun..

[41] Demion M., Bois P., Launay P., Guinamard R. (2007). TRPM4, a Ca^2+^-activated nonselective cation channel in mouse sino-atrial node cells. Cardiovasc. Res..

[42] Hof T., Simard C., Rouet R., Salle L., Guinamard R. (2013). Implication of the TRPM4 nonselective cation channel in mammalian sinus rhythm. Heart Rhythm.

[43] Kapoor N., Tran A., Kang J., Zhang R., Philipson K.D., Goldhaber J.I. (2015). Regulation of calcium clock-mediated pacemaking by inositol-1,4,5-trisphosphate receptors in mouse sinoatrial nodal cells. J. Physiol..

[44] Torrente A.G., Zhang R., Wang H., Zaini A., Kim B., Yue X., Philipson K.D., Goldhaber J.I. (2017). Contribution of small conductance K(^+^) channels to sinoatrial node pacemaker activity: Insights from atrial-specific Na(^+^)/Ca(^2+^) exchange knockout mice. J. Physiol..

[45] Yaniv Y., Spurgeon H.A., Lyashkov A.E., Yang D.M., Ziman B.D., Maltsev V.A., Lakatta E.G. (2012). Crosstalk between Mitochondrial and Sarcoplasmic Reticulum Ca^2+^ Cycling Modulates Cardiac Pacemaker Cell Automaticity. PLoS ONE.

[46] Yaniv Y., Spurgeon H.A., Ziman B.D., Lyashkov A.E., Lakatta E.G. (2013). Mechanisms that match ATP supply to demand in cardiac pacemaker cells during high ATP demand. Am. J. Physiol.-Heart Circ. Physiol..

[47] Allah E.A., Tellez J.O., Yanni J., Nelson T., Monfredi O., Boyett M.R., Dobrzynski H. (2011). Changes in the expression of ion channels, connexins and Ca^2+^-handling proteins in the sino-atrial node during postnatal development. Exp. Physiol..

[48] Chandler N.J., Greener I.D., Tellez J.O., Inada S., Musa H., Molenaar P., DiFrancesco D., Baruscotti M., Longhi R., Anderson R.H. (2009). Molecular Architecture of the Human Sinus Node Insights Into the Function of the Cardiac Pacemaker. Circulation.

[49] Marionneau C., Couette B., Liu J., Li H., Mangoni M.E., Nargeot J., Lei M., Escande D., Demolombe S. (2005). Specific pattern of ionic channel gene expression associated with pacemaker activity in the mouse heart. J. Physiol..

[50] Musa H., Lei M., Honjo H., Jones S.A., Dobrzynski H., Lancaster M.K., Takagishi Y., Henderson Z., Kodama I., Boyett M.R. (2002). Heterogeneous expression of Ca(^2+^) handling proteins in rabbit sinoatrial node. J. Histochem. Cytochem..

[51] Satoh H. (2003). Sino-atrial nodal cells of mammalian hearts: Ionic currents and gene expression of pacemaker ionic channels. J. Smooth Muscle Res..

[52] Tellez J.O., Dobrzynski H., Greener I.D., Graham G.M., Laing E., Honjo H., Hubbard S.J., Boyett M.R., Billeter R. (2006). Differential expression of ion channel transcripts in atrial muscle and sinoatrial node in rabbit. Circ. Res..

[53] Duan D., Fermini B., Nattel S. (1993). Potassium channel blocking properties of propafenone in rabbit atrial myocytes. J. Pharmacol. Exp. Ther..

[54] Klimek-Piotrowska W., Krawczyk-Ozog A., Suski M., Kapusta P., Wolkow P.P., Holda M.K. (2018). Comparative iTRAQ analysis of protein abundance in the human sinoatrial node and working cardiomyocytes. J. Anat..

[55] Christoffels V.M., Smits G.J., Kispert A., Moorman A.F. (2010). Development of the pacemaker tissues of the heart. Circ. Res..

[56] Gourdie R.G., Harris B.S., Bond J., Justus C., Hewett K.W., O’Brien T.X., Thompson R.P., Sedmera D. (2003). Development of the cardiac pacemaking and conduction system. Birth Defects Res. C Embryo Today.

[57] Mommersteeg M.T., Hoogaars W.M., Prall O.W., de Gier-de Vries C., Wiese C., Clout D.E., Papaioannou V.E., Brown N.A., Harvey R.P., Moorman A.F. (2007). Molecular pathway for the localized formation of the sinoatrial node. Circ. Res..

[58] Wiese C., Grieskamp T., Airik R., Mommersteeg M.T., Gardiwal A., de Gier-de Vries C., Schuster-Gossler K., Moorman A.F., Kispert A., Christoffels V.M. (2009). Formation of the sinus node head and differentiation of sinus node myocardium are independently regulated by Tbx18 and Tbx3. Circ. Res..

[59] Cheng H., Lederer W.J., Cannell M.B. (1993). Calcium sparks: Elementary events underlying excitation-contraction coupling in heart muscle. Science.

[60] Verkerk A.O., van Borren M.M., Wilders R. (2013). Calcium transient and sodium-calcium exchange current in human versus rabbit sinoatrial node pacemaker cells. ScientificWorldJournal.

[61] Lyashkov A.E., Juhaszova M., Dobrzynski H., Vinogradova T.M., Maltsev V.A., Juhasz O., Spurgeon H.A., Sollott S.J., Lakatta E.G. (2007). Calcium cycling protein density and functional importance to automaticity of isolated sinoatrial nodal cells are independent of cell size. Circ. Res..

[62] Stern M.D., Maltseva L.A., Juhaszova M., Sollott S.J., Lakatta E.G., Maltsev V.A. (2014). Hierarchical clustering of ryanodine receptors enables emergence of a calcium clock in sinoatrial node cells. J. Gen. Physiol..

[63] Lukyanenko V., Gyorke I., Subramanian S., Smirnov A., Wiesner T.F., Gyorke S. (2000). Inhibition of Ca^2+^ sparks by ruthenium red in permeabilized rat ventricular myocytes. Biophys. J..

[64] Lukyanenko V., Gyorke S. (1999). Ca^2+^ sparks and Ca^2+^ waves in saponin-permeabilized rat ventricular myocytes. J. Physiol..

[65] Zima A.V., Bovo E., Bers D.M., Blatter L.A. (2010). Ca^2+^ spark-dependent and -independent sarcoplasmic reticulum Ca^2+^ leak in normal and failing rabbit ventricular myocytes. J. Physiol..

[66] Sirenko S., Maltsev V.A., Maltseva L.A., Yang D., Lukyanenko Y., Vinogradova T.M., Jones L.R., Lakatta E.G. (2014). Sarcoplasmic reticulum Ca cycling protein phosphorylation in a physiologic Ca milieu unleashes a high-power, rhythmic Ca clock in ventricular myocytes: Relevance to arrhythmias and bio-pacemaker design. J. Mol. Cell. Cardiol..

[67] Fabiato A. (1985). Time and calcium dependence of activation and inactivation of calcium-induced release of calcium from the sarcoplasmic reticulum of a skinned canine cardiac Purkinje cell. J. Gen. Physiol..

[68] Bassani J.W., Bassani R.A., Bers D.M. (1995). Calibration of indo-1 and resting intracellular [Ca]i in intact rabbit cardiac myocytes. Biophys. J..

[69] Maltsev A.V., Maltsev V.A., Mikheev M., Maltseva L.A., Sirenko S.G., Lakatta E.G., Stern M.D. (2011). Synchronization of stochastic Ca^2+^ release units creates a rhythmic Ca^2+^ clock in cardiac pacemaker cells. Biophys. J..

[70] Maxwell J.T., Blatter L.A. (2017). A novel mechanism of tandem activation of ryanodine receptors by cytosolic and SR luminal Ca(^2+^) during excitation-contraction coupling in atrial myocytes. J. Physiol..

[71] Periasamy M., Bhupathy P., Babu G.J. (2008). Regulation of sarcoplasmic reticulum Ca^2+^ ATPase pump expression and its relevance to cardiac muscle physiology and pathology. Cardiovasc. Res..

[72] Frank K.F., Bolck B., Erdmann E., Schwinger R.H. (2003). Sarcoplasmic reticulum Ca^2+^-ATPase modulates cardiac contraction and relaxation. Cardiovasc. Res..

[73] Luss I., Boknik P., Jones L.R., Kirchhefer U., Knapp J., Linck B., Luss H., Meissner A., Muller F.U., Schmitz W. (1999). Expression of cardiac calcium regulatory proteins in atrium v ventricle in different species. J. Mol. Cell. Cardiol..

[74] Minajeva A., Kaasik A., Paju K., Seppet E., Lompre A.M., Veksler V., Ventura-Clapier R. (1997). Sarcoplasmic reticulum function in determining atrioventricular contractile differences in rat heart. Am. J. Physiol..

[75] Boknik P., Unkel C., Kirchhefer U., Kleideiter U., Klein-Wiele O., Knapp J., Linck B., Luss H., Muller F.U., Schmitz W. (1999). Regional expression of phospholamban in the human heart. Cardiovasc. Res..

[76] Eisner D., Bode E., Venetucci L., Trafford A. (2013). Calcium flux balance in the heart. J. Mol. Cell. Cardiol..

[77] Goeger D.E., Riley R.T., Dorner J.W., Cole R.J. (1988). Cyclopiazonic acid inhibition of the Ca^2+^-transport ATPase in rat skeletal muscle sarcoplasmic reticulum vesicles. Biochem. Pharmacol..

[78] Kranias E.G., Hajjar R.J. (2012). Modulation of Cardiac Contractility by the Phopholamban/SERCA2a Regulatome. Circ. Res..

[79] Baker D.L., Hashimoto K., Grupp I.L., Ji Y., Reed T., Loukianov E., Grupp G., Bhagwhat A., Hoit B., Walsh R. (1998). Targeted overexpression of the sarcoplasmic reticulum Ca^2+^-ATPase increases cardiac contractility in transgenic mouse hearts. Circ. Res..

[80] Andersson K.B., Birkeland J.A., Finsen A.V., Louch W.E., Sjaastad I., Wang Y., Chen J., Molkentin J.D., Chien K.R., Sejersted O.M. (2009). Moderate heart dysfunction in mice with inducible cardiomyocyte-specific excision of the Serca2 gene. J. Mol. Cell. Cardiol..

[81] Louch W.E., Hougen K., Mork H.K., Swift F., Aronsen J.M., Sjaastad I., Reims H.M., Roald B., Andersson K.B., Christensen G. (2010). Sodium accumulation promotes diastolic dysfunction in end-stage heart failure following Serca2 knockout. J. Physiol..

[82] Cain B.S., Meldrum D.R., Joo K.S., Wang J.F., Meng X., Cleveland J.C., Banerjee A., Harken A.H. (1998). Human SERCA2a levels correlate inversely with age in senescent human myocardium. J. Am. Coll. Cardiol..

[83] Lakatta E.G. (1993). Myocardial adaptations in advanced age. Basic Res. Cardiol..

[84] Lompre A.M., Lambert F., Lakatta E.G., Schwartz K. (1991). Expression of sarcoplasmic reticulum Ca(^2+^)-ATPase and calsequestrin genes in rat heart during ontogenic development and aging. Circ. Res..

[85] Liu J., Sirenko S., Juhaszova M., Sollott S.J., Shukla S., Yaniv Y., Lakatta E.G. (2014). Age-associated abnormalities of intrinsic automaticity of sinoatrial nodal cells are linked to deficient cAMP-PKA-Ca(^2+^) signaling. Am. J. Physiol. Heart Circ. Physiol..

[86] Arai M., Alpert N.R., Maclennan D.H., Barton P., Periasamy M. (1993). Alterations in Sarcoplasmic-Reticulum Gene-Expression in Human Heart-Failure—A Possible Mechanism for Alterations in Systolic and Diastolic Properties of the Failing Myocardium. Circ. Res..

[87] Arai M., Matsui H., Periasamy M. (1994). Sarcoplasmic reticulum gene expression in cardiac hypertrophy and heart failure. Circ. Res..

[88] Hasenfuss G., Meyer M., Schillinger W., Preuss M., Pieske B., Just H. (1997). Calcium handling proteins in the failing human heart. Basic Res. Cardiol..

[89] Hasenfuss G. (1998). Alterations of calcium-regulatory proteins in heart failure. Cardiovasc. Res..

[90] Winslow R.L., Rice J., Jafri S., Marban E., O’Rourke B. (1999). Mechanisms of altered excitation-contraction coupling in canine tachycardia-induced heart failure, II: Model studies. Circ. Res..

[91] Periasamy M., Huke S. (2001). SERCA pump level is a critical determinant of Ca^2+^ homeostasis and cardiac contractility. J. Mol. Cell. Cardiol..

[92] Sakata S., Lebeche D., Sakata N., Sakata Y., Chemaly E.R., Liang L.F., Tsuji T., Takewa Y., del Monte F., Peluso R. (2007). Restoration of mechanical and energetic function in failing aortic-banded rat hearts by gene transfer of calcium cycling proteins. J. Mol. Cell. Cardiol..

[93] Beeri R., Chaput M., Guerrero J.L., Kawase Y., Yosefy C., Abedat S., Karakikes I., Morel C., Tisosky A., Sullivan S. (2010). Gene delivery of sarcoplasmic reticulum calcium ATPase inhibits ventricular remodeling in ischemic mitral regurgitation. Circ. Heart Fail..

[94] Mariani J.A., Smolic A., Preovolos A., Byrne M.J., Power J.M., Kaye D.M. (2011). Augmentation of left ventricular mechanics by recirculation-mediated AAV2/1-SERCA2a gene delivery in experimental heart failure. Eur. J. Heart Fail..

[95] Papolos A., Frishman W.H. (2013). Sarcoendoplasmic reticulum calcium transport ATPase 2a: A potential gene therapy target in heart failure. Cardiol. Rev..

[96] Laraia P.J., Morkin E. (1974). Adenosine 3′,5′-Monophosphate-Dependent Membrane Phosphorylation—Possible Mechanism for Control of Microsomal Calcium-Transport in Heart-Muscle. Circ. Res..

[97] MacLennan D.H., Kranias E.G. (2003). Phospholamban: A crucial regulator of cardiac contractility. Nat. Rev. Mol. Cell. Biol..

[98] Luo W., Wolska B.M., Grupp I.L., Harrer J.M., Haghighi K., Ferguson D.G., Slack J.P., Grupp G., Doetschman T., Solaro R.J. (1996). Phospholamban gene dosage effects in the mammalian heart. Circ. Res..

[99] Santana L.F., Kranias E.G., Lederer W.J. (1997). Calcium sparks and excitation-contraction coupling in phospholamban-deficient mouse ventricular myocytes. J. Physiol..

[100] Koss K.L., Grupp I.L., Kranias E.G. (1997). The relative phospholamban and SERCA2 ratio: A critical determinant of myocardial contractility. Basic Res. Cardiol..

[101] Walden A.P., Dibb K.M., Trafford A.W. (2009). Differences in intracellular calcium homeostasis between atrial and ventricular myocytes. J. Mol. Cell. Cardiol..

[102] Wolska B.M., Stojanovic M.O., Luo W., Kranias E.G., Solaro R.J. (1996). Effect of ablation of phospholamban on dynamics of cardiac myocyte contraction and intracellular Ca^2+^. Am. J. Physiol..

[103] Del Monte F., Harding S.E., Dec G.W., Gwathmey J.K., Hajjar R.J. (2002). Targeting phospholamban by gene transfer in human heart failure. Circulation.

[104] Haghighi K., Kolokathis F., Gramolini A.O., Waggoner J.R., Pater L., Lynch R.A., Fan G.C., Tsiapras D., Parekh R.R., Dorn G.W. (2006). A mutation in the human phospholamban gene, deleting arginine 14, results in lethal, hereditary cardiomyopathy. Proc. Natl. Acad. Sci. USA.

[105] Haghighi K., Kolokathis F., Pater L., Lynch R.A., Asahi M., Gramolini A.O., Fan G.C., Tsiapras D., Hahn H.S., Adamopoulos S. (2003). Human phospholamban null results in lethal dilated cardiomyopathy revealing a critical difference between mouse and human. J. Clin. Investig..

[106] Vinogradova T.M., Lyashkov A.E., Zhu W., Ruknudin A.M., Sirenko S., Yang D., Deo S., Barlow M., Johnson S., Caffrey J.L. (2006). High basal protein kinase A-dependent phosphorylation drives rhythmic internal Ca^2+^ store oscillations and spontaneous beating of cardiac pacemaker cells. Circ. Res..

[107] Katsushika S., Chen L., Kawabe J., Nilakantan R., Halnon N.J., Homcy C.J., Ishikawa Y. (1992). Cloning and characterization of a sixth adenylyl cyclase isoform: Types V and VI constitute a subgroup within the mammalian adenylyl cyclase family. Proc. Natl. Acad. Sci. USA.

[108] Premont R.T., Chen J., Ma H.W., Ponnapalli M., Iyengar R. (1992). Two members of a widely expressed subfamily of hormone-stimulated adenylyl cyclases. Proc. Natl. Acad. Sci. USA.

[109] Yoshimura M., Cooper D.M.F. (1992). Cloning and Expression of a Ca^2+^-Inhibitable Adenylyl Cyclase from Ncb-20 Cells. Proc. Natl. Acad. Sci. USA.

[110] Mattick P., Parrington J., Odia E., Simpson A., Collins T., Terrar D. (2007). Ca^2+^-stimulated adenylyl cyclase isoform AC1 is preferentially expressed in guinea-pig sino-atrial node cells and modulates the I_f_ pacemaker current. J. Physiol..

[111] Younes A., Lyashkov A.E., Graham D., Sheydina A., Volkova M.V., Mitsak M., Vinogradova T.M., Lukyanenko Y.O., Li Y., Ruknudin A.M. (2008). Ca^2+^ -stimulated basal adenylyl cyclase activity localization in membrane lipid microdomains of cardiac sinoatrial nodal pacemaker cells. J. Biol. Chem..

[112] Vinogradova T.M., Zhou Y.Y., Bogdanov K.Y., Yang D., Kuschel M., Cheng H., Xiao R.P. (2000). Sinoatrial node pacemaker activity requires Ca(^2+^)/calmodulin-dependent protein kinase II activation. Circ. Res..

[113] Vinogradova T.M., Sirenko S., Lyashkov A.E., Younes A., Li Y., Zhu W., Yang D., Ruknudin A.M., Spurgeon H., Lakatta E.G. (2008). Constitutive phosphodiesterase activity restricts spontaneous beating rate of cardiac pacemaker cells by suppressing local Ca^2+^ releases. Circ. Res..

[114] Kranias E.G., Solaro R.J. (1982). Phosphorylation of troponin I and phospholamban during catecholamine stimulation of rabbit heart. Nature.

[115] Lindemann J.P., Jones L.R., Hathaway D.R., Henry B.G., Watanabe A.M. (1983). beta-Adrenergic stimulation of phospholamban phosphorylation and Ca^2+^-ATPase activity in guinea pig ventricles. J. Biol. Chem..

[116] Garvey J.L., Kranias E.G., Solaro R.J. (1988). Phosphorylation of C-protein, troponin I and phospholamban in isolated rabbit hearts. Biochem. J..

[117] Wegener A.D., Simmerman H.K., Lindemann J.P., Jones L.R. (1989). Phospholamban phosphorylation in intact ventricles. Phosphorylation of serine 16 and threonine 17 in response to beta-adrenergic stimulation. J. Biol. Chem..

[118] Talosi L., Edes I., Kranias E.G. (1993). Intracellular mechanisms mediating reversal of beta-adrenergic stimulation in intact beating hearts. Am. J. Physiol..

[119] MundinaWeilenmann C., Vittone L., Ortale M., deCingolani G.C., Mattiazzi A. (1996). Immunodetection of phosphorylation sites gives new insights into the mechanisms underlying phospholamban phosphorylation in the intact heart. J. Biol. Chem..

[120] Gomez A.M., Cheng H.P., Lederer W.J., Bers D.M. (1996). Ca^2+^ diffusion and sarcoplasmic reticulum transport both contribute to [Ca^2+^](i) decline during Ca^2+^ sparks in rat ventricular myocytes. J. Physiol..

[121] Guo T., Zhang T., Ginsburg K.S., Mishra S., Brown J.H., Bers D.M. (2012). CaMKII delta(C) Slows [Ca](i) Decline in Cardiac Myocytes by Promoting Ca Sparks. Biophys. J..

[122] Currie S., Loughrey C.M., Craig M.A., Smith G.L. (2004). Calcium/calmodulin-dependent protein kinase II delta associates with the ryanodine receptor complex and regulates channel function in rabbit heart. Biochem. J..

[123] Maier L.S., Bers D.M. (2007). Role of Ca^2+^/calmodulin-dependent protein kinase (CaMK) in excitation-contraction coupling in the heart. Cardiovasc. Res..

[124] Wehrens X.H., Lehnart S.E., Reiken S.R., Marks A.R. (2004). Ca^2+^/calmodulin-dependent protein kinase II phosphorylation regulates the cardiac ryanodine receptor. Circ. Res..

[125] Curran J., Hinton M.J., Rios E., Bers D.M., Shannon T.R. (2007). Beta-adrenergic enhancement of sarcoplasmic reticulum calcium leak in cardiac myocytes is mediated by calcium/calmodulin-dependent protein kinase. Circ. Res..

[126] Shan J., Kushnir A., Betzenhauser M.J., Reiken S., Li J., Lehnart S.E., Lindegger N., Mongillo M., Mohler P.J., Marks A.R. (2010). Phosphorylation of the ryanodine receptor mediates the cardiac fight or flight response in mice. J. Clin. Investig..

[127] Petit-Jacques J., Bois P., Bescond J., Lenfant J. (1993). Mechanism of muscarinic control of the high-threshold calcium current in rabbit sino-atrial node myocytes. Pflugers Arch..

[128] Zhou P., Zhao Y.T., Guo Y.B., Xu S.M., Bai S.H., Lakatta E.G., Cheng H., Hao X.M., Wang S.Q. (2009). Beta-adrenergic signaling accelerates and synchronizes cardiac ryanodine receptor response to a single L-type Ca^2+^ channel. Proc. Natl. Acad. Sci. USA.

[129] Zhang R., Khoo M.S., Wu Y., Yang Y., Grueter C.E., Ni G., Price E.E., Thiel W., Guatimosim S., Song L.S. (2005). Calmodulin kinase II inhibition protects against structural heart disease. Nat. Med..

[130] Xu L., Lai D., Cheng J., Lim H.J., Keskanokwong T., Backs J., Olson E.N., Wang Y. (2010). Alterations of L-type calcium current and cardiac function in CaMKII{delta} knockout mice. Circ. Res..

[131] Gao Z., Singh M.V., Hall D.D., Koval O.M., Luczak E.D., Joiner M.L., Chen B., Wu Y., Chaudhary A.K., Martins J.B. (2011). Catecholamine-independent heart rate increases require Ca^2+^/calmodulin-dependent protein kinase II. Circ. Arrhythm. Electrophysiol..

[132] Collins T.P., Terrar D.A. (2012). Ca(^2+^)-stimulated adenylyl cyclases regulate the L-type Ca(^2+^) current in guinea-pig atrial myocytes. J. Physiol..

[133] De Koninck P., Schulman H. (1998). Sensitivity of CaM kinase II to the frequency of Ca^2+^ oscillations. Science.

[134] Rich R.C., Schulman H. (1998). Substrate-directed function of calmodulin in autophosphorylation of Ca^2+^/calmodulin-dependent protein kinase II. J. Biol. Chem..

[135] Ginsburg K.S., Weber C.R., Bers D.M. (1998). Control of maximum sarcoplasmic reticulum Ca load in intact ferret ventricular myocytes. Effects Of thapsigargin and isoproterenol. J. Gen. Physiol..

[136] Akin B.L., Chen Z., Jones L.R. (2010). Superinhibitory phospholamban mutants compete with Ca^2+^ for binding to SERCA2a by stabilizing a unique nucleotide-dependent conformational state. J. Biol. Chem..

[137] Akin B.L., Jones L.R. (2012). Characterizing phospholamban to sarco(endo)plasmic reticulum Ca^2+^-ATPase 2a (SERCA2a) protein binding interactions in human cardiac sarcoplasmic reticulum vesicles using chemical cross-linking. J. Biol. Chem..

